# Characterization of Two Novel Heat Shock Protein 70 Transcripts from *Sitodiplosis mosellana* and Their Response to Larval Diapause and Thermal Stress

**DOI:** 10.3390/biology14091147

**Published:** 2025-08-30

**Authors:** Qitong Huang, Wenqian Tang, Xiaobin Liu, Qian Ma, Keyan Zhu-Salzman, Weining Cheng

**Affiliations:** 1 Shandong Institute of Sericulture, Shandong Academy of Agricultural Sciences, Yantai 265503, China; 13760637249hqt@nwafu.edu.cn (Q.H.); wenqiantang@126.com (W.T.); bin0331@126.com (X.L.); 2 Key Laboratory of Plant Protection Resources and Pest Management of Ministry of Education, College of Plant Protection, Northwest A&F University, Yangling 712100, China; maqian981017@163.com; 3 Department of Entomology, Texas A&M University, College Station, TX 77843, USA

**Keywords:** *Sitodiplosis mosellana*, heat shock protein, diapause, stress response, cold resistance

## Abstract

The heat shock protein 70 (Hsp70) family mediates responses to environmental stress in insects. The wheat midge *Sitodiplosis mosellana*, a worldwide pest, avoids summer and winter temperature extremes by diapause of the third-instar larvae in the soil. To explore the functions of Hsp70s in this process, we characterized two cytoplasmic Hsp70 genes (*SmHsp70A1-1* and *SmHsp70A1-2*) from this insect. Both SmHsp70s contained three signature motifs of the family and lacked introns. Developmental expression profiling revealed maximal *SmHsp70A1-1* expression during early larval stages, while the expression of *SmHsp70A1-2* was highest in the pupal stages. The expression of *SmHsp70A1-1* was significantly upregulated during diapause, particularly during summer and winter, whereas *SmHsp70A1-2* showed marked downregulation and dose-dependent induction by 20-hydroxyecdysone (20E). Furthermore, both genes exhibited similar expression patterns in over-summering and over-wintering larvae under thermal stress, with maximal expression at 40 °C and −10 °C, respectively, but were not significantly induced at prolonged extreme temperatures (50 °C or −15 °C). Knockdown of the two *SmHsp70* genes by RNA interference (RNAi) significantly increased the susceptibility of the larvae to cold stress. These results suggest the important role of both *SmHsp70* genes in diapause-associated stress tolerance and provide crucial insights into the mechanisms underlying thermal adaptation in *S. mosellana*.

## Simple Summary

The heat shock protein 70 (Hsp70) family plays key roles not only in heat/cold stress tolerance but also in insect diapause. In this study, we characterized two cytoplasmic Hsp70 genes (*SmHsp70A1-1* and *SmHsp70A1-2*) from *Sitodiplosis mosellana*, a major wheat pest undergoing obligatory diapause as third-instar larvae in the soil, and analyzed their expression profiles and functions in relation to diapause and thermal stress. The results showed that the expression of *SmHsp70A1-1* and *SmHsp70A1-2* is developmentally and environmentally regulated, with increased expression being essential for enhancing cold tolerance in *S. mosellana*. The findings provide crucial insights into the mechanisms underlying thermal tolerance in this pest.

## 1. Introduction

Heat shock proteins (Hsps) are a superfamily of molecular chaperones whose expression is upregulated under stress conditions [1,2]. They were originally discovered in connection with the heat shock response in *Drosophila* [3] but are now recognized as being inducible by various stressors, including cold shock, chemical pesticides, and pathogenic invasion [4,5,6]. The primary function of Hsps is to preserve proteostasis by preventing aberrant protein aggregation and promoting clearance of misfolded or aggregated proteins [7,8]. Six distinct Hsp families are recognized based on evolutionary lineage and molecular weight, namely, the Hsp100, Hsp90, Hsp70, Hsp60, Hsp40, and small Hsp families [9,10,11].

Among Hsps, Hsp70s represent a group of structurally and evolutionarily conserved multifunctional proteins that fall into two subtypes, namely, the stress-inducible heat shock protein (Hsp70) and heat shock cognate protein 70 (Hsc70) subtypes [12,13]. The mRNA expression of Hsp70s is induced rapidly from basal levels in response to various stress stimuli, whereas Hsc70 proteins are expressed constitutively at relatively high levels with minimal variation under stress conditions [14,15]. Both protein types are approximately 70 kDa in molecular weight and share several common structural features. In insects, Hsp70s are suggested to participate in multiple physiological processes, including enhancing resistance to extreme temperatures to protect against thermal stress-induced injury and death [16,17]. In addition, Hsp70s have been shown to modulate larval development in *Spodoptera litura*, with their expression being induced by ecdysone (20-hydroxyecdysone [20E]) [18].

An emerging body of evidence also indicates the involvement of Hsp70s in insect diapause, an adaptive physiological tactic employed by insects to withstand adverse environmental conditions, including extreme temperatures associated with seasonal changes [19,20]. For instance, in the cabbage butterfly *Pieris melete*, expression of *PmHsp70b* is induced during diapause, whereas that of *PmHsp70a* is unaffected [21]. Similarly, increased expression of *Hsp70* genes during diapause has been reported in various insect taxa, such as *Sarcophaga crassipalpis Hsp70a* and *Hsp70b* [22], *Leptinotarsa decemlineata Hsp70a* [23], *Bombyx mori Hsp70a* [24], and *Ostrinia nubilalis Hsp70* [25]. In contrast, *Hsp70* expression is either downregulated or maintained at low levels during diapause in *Omphisa fuscidentalis* [26], *Sesamia nonagrioides* [27], and *Helicoverpa zea* [28]. These variations in expression suggest that the functions of *Hsp70s* during diapause differ according to the insect species.

The orange wheat blossom midge, *Sitodiplosis mosellana* Géhin (Diptera: Cecidomyiidae), is a cosmopolitan and destructive pest of wheat crops, causing significant reductions in yield and economic repercussions during outbreak years [29,30,31]. Typically, this species completes a univoltine life cycle annually. In most wheat-producing regions of northern China, adult emergence and subsequent egg-laying take place from late April to early May [32]. Upon hatching, the larvae feed on the developing grains until the third instar. By mid-to-late May, the mature larvae exit the wheat heads and descend to the ground, where they burrow into the soil to spin cocoons for obligatory diapause. Although the larvae remain inside their protective cocoons until mid-March of the following year, diapause is effectively terminated by low temperatures in early December [33]. The larvae then enter a quiescent post-diapause stage that does not differ morphologically from diapause. The initiation of post-diapausal development is triggered by the rise in ambient temperature during the spring, prompting the larvae to exit their cocoons and proceed to pupation on the soil surface. These prolonged periods of diapause and quiescence thus shield the pest from seasonal temperature extremes, enabling synchronization of its lifecycle with wheat phenology.

In our previous studies, an *Hsp70* gene from *S. mosellana* was identified and characterized, and its expression patterns during diapause were elucidated [34]. Despite this, the potential involvement and functional roles of other Hsp70 family members in the diapause response remain unknown. In this study, we identified two additional *Hsp70* genes (*SmHsp70A1-1* and *SmHsp70A1-2*) in *S. mosellana* larvae and analyzed their transcriptional profiles during direct development and diapause, as well as their responsiveness to extreme temperatures or 20E treatment. Furthermore, RNA interference (RNAi) was used to knock down the expression of these two *SmHsp70* genes and assess cold tolerance between the knockdown larvae and the control group. The findings provide crucial insights into the molecular processes underlying stress resistance in diapausing *S. mosellana*.

## 2. Materials and Methods

### 2.1. Insect Source

*S. mosellana* individuals at all post-embryonic stages (excluding eggs) were collected from natural habitats using an established method [35]. Specifically, first- and second-instar larvae (n = 50) and pre-diapausing third-instar larvae (n = 20), characterized by phenotypic traits (coloration and body size) and the presence of a Y-shaped sclerite, were obtained through the dissection of *S. mosellana*-infested wheat spikes during the grain-filling period in May 2022. Concurrently, large amounts of wheat spikes infested with third-stage larvae were pooled and translocated to soil within an experimental field insectary in Yangling, China (34°16′ N, 108°4′ E). Soil moisture was maintained through intermittent watering to stimulate diapause initiation and termination. Successful transition into diapause was confirmed by the formation of larval cocoons. Cocooned larvae at the diapause (June–November, 2022) and post-diapause quiescent (December 2022–February 2023) phases were collected monthly by sieving the soil in the field insectary. Post-diapause developing larvae, pre-pupae, early-, mid-, and late-stage pupae, and adults were collected in succession from mid-March onward. All collected samples were immediately frozen in liquid nitrogen, followed by storage at −80 °C for later analyses.

### 2.2. RNA Extraction, cDNA Synthesis, and gDNA Isolation

Total RNA was isolated from pre-diapause *S. mosellana* larvae (n = 20) using the RNAsimple Total RNA Kit (Tiangen, Beijing, China) according to the manufacturer’s protocol. RNA integrity was checked by electrophoresis on 1% agarose gels and quantified using the BioSpec-nano spectrophotometer (Shimadzu, Kyoto, Japan). High-quality RNA (1 μg) was then reverse-transcribed to cDNA using the PrimeScript™ RT Reagent Kit with gDNA Eraser (TaKaRa, Dalian, China). For the isolation of genomic DNA, pooled larval samples (n = 20) were processed with the Biospin Insect Genomic DNA Extraction Kit (Bioer Technology Co., Ltd., Hangzhou, China), as directed.

### 2.3. Gene Cloning

Primers targeting the open reading frames (ORFs) of *SmHsp70A1-1-F/R* and *Sm70A1-2-F/R* (Table 1) were designed based on transcriptome sequencing data from *S. mosellana* larvae. For amplification, each 25 µL reaction volume contained 12.5 μL of PrimeSTAR Max Premix (Takara, Beijing, China), 1.0 μL of cDNA template, 1.0 μL of each specific primer (10 μM), and 9.5 μL of RNase-free water. The thermal cycling conditions used for PCR are as follows: 38 cycles at 98 °C for 10 s, 54 °C for 15 s, and 72 °C for 40 s. The PCR amplicons were gel-purified using a gel extraction kit (CWBIO, Beijing, China). The purified DNAs were cloned into a pEASY^®^-Blunt Zero vector and transferred into *Trans1-T1* competent cells (TransGen, Beijing, China). Three positive clones per PCR amplicon were randomly selected for sequencing by AuGCT DNA-SYN Biotech (Beijing, China).

### 2.4. Bioinformatic Analysis of SmHsp70s

The molecular weights and isoelectric points of the SmHsp70A1-1 and SmHsp70A1-2 proteins were calculated using the Compute pI/Mw tool (https://web.expasy.org/compute_pi/, accessed on 10 March 2023). Conserved functional domains in the deduced protein sequences were identified using the NCBI Conserved Domain Database (https://www.ncbi.nlm.nih.gov/Structure/cdd/wrpsb.cgi, accessed on 10 March 2023). Subcellular localization of the proteins was predicted using the CELLO v2.5 program (http://cello.life.nctu.edu.tw/, accessed on 10 March 2023). Multiple sequence alignments of *S. mosellana* Hsp70A1-1 and Hsp70A1-2 with their homologs from other insects were performed using DNAMAN v.7.0.2 (Lynnon Biosoft, San Ramon, CA, USA). A neighbor-joining phylogenetic tree was constructed using MEGA X version 10.0.1 (Temple University, Philadelphia, PA, USA) with 1000 bootstrap replicates, excluding bootstrap values less than 50.

### 2.5. 20E Treatment

To investigate the influence of 20E on the transcriptional levels of the two *SmHsp70* genes in diapausing larvae, 23 nl of 20E (0.1–0.6 pg/nL in 50% ethanol; Sigma-Aldrich, St. Louis, MO, USA) was injected into the abdomens of each October-collected cocooned larvae using a Nanoject II microinjection device (Drummond Scientific Company, Broomall, PA, USA). These larval stages were chosen due to their low expression of *SmHsp70A1-1* and *SmHsp70A1-2* (refer to Section 3). The doses used for injection reflected the endogenous 20E levels reported for this species [36]. Following injection, the larvae were maintained in Petri dishes containing moist filter paper at 25 °C for 3, 6, or 12 h in a controlled climate chamber, followed by flash-freezing in liquid nitrogen and storage at −80 °C for subsequent RNA extraction. The control groups consisted of insects treated with an equivalent volume of 50% ethanol. Each treatment was replicated three times with at least 20 individuals per replicate.

### 2.6. Temperature Treatments

*Sitodiplosis mosellana* typically undergoes diapause as cocooned larvae at a soil depth of 3–10 cm to survive summer and winter [37]. In the Yangling zone, Shaanxi Province, China (34°16′ N, 108°4′ E), this soil layer is subjected to maximum temperatures of around 35 °C in summer and minimum temperatures close to 0 °C in winter. However, surface extremes routinely exceed 50 °C in summer and plunge to −15 °C in winter [38]. To clarify the prospective roles of *SmHsp70A1-1* and *SmHsp70A1-2* under these circumstances, over-summering and over-wintering larvae were subjected to short-term heat and cold treatments. In brief, 20 August-collected cocooned larvae or December-collected cocooned larvae were placed in sterile cryotubes. To elicit a heat shock response, the tubes containing August-collected larvae were submerged in water baths at varying temperatures (35–50 °C in increments of 5 °C) for 1 h and at temperatures of 40 °C and 45 °C for different durations (15–120 min). The December-collected larvae were treated in a similar way, with incubation in temperature-controlled chambers at varying temperatures (−15–0 °C) for 1 h and at −5 °C and −10 °C for different durations (15–120 min). Larvae collected before the temperature treatments were used as the controls. At each time point post-treatment, the larvae were flash-frozen in liquid nitrogen and stored at −80 °C for analysis by qRT-PCR. All treatments were replicated a minimum of three times.

### 2.7. Quantitative Real-Time Polymerase Chain Reaction (qRT-PCR)

To examine the developmental expression profiles of the two *SmHsp70* genes, total RNA was isolated from first- to second-instar larvae (n = 50), third-instar larvae at each diapause stage (n = 20, per stage), pupae at early, mid-, and late developmental stages (n = 20, per stage), and adult females and males (n = 20, per sex). For the analysis of transcript abundance in 20E-treated larvae, RNA was isolated from 20 individuals per treatment group. Total RNA isolation and cDNA synthesis were performed as described above.

qRT-PCR was performed with SuperReal PreMix Plus (SYBR Green) (Tiangen, Beijing, China) on a Bio-Rad CFX96 PCR system (Hercules, CA, USA). Each reaction was made up to 20 µL, comprising 10 μL of 2 × SuperReal PreMix Plus (Tiangen, Beijing, China), 1.0 μL of cDNA template, 1.2 μL of primer mix (10 μM of each sense and antisense primer) (Table 1), and 7.8 μL of double-distilled water. The following cycling conditions were used: 95 °C/30 s; 35 cycles of 95 °C/5 s; and 60 °C/30 s. A melting curve analysis was then conducted for each reaction from 60 to 95 °C to assess the specificity and homogeneity of the PCR products. RNase-free water replaced the cDNA template in no-template control reactions to confirm assay specificity and validity. *Gapdh* (GenBank accession number: KR733066) was used as the internal reference for normalization. Each sample underwent three independent tests with three replicates, and the relative expression levels of each transcript were quantified using the 2^−ΔΔCT^ method.

### 2.8. Double-Stranded RNA (dsRNA) Synthesis and Delivery

dsRNA primers incorporating a T7 promoter sequence (TAATACGACTCACTATAGGG) at the 5′ end of both the forward and reverse strands were designed using the SnapDragon dsRNA Design tool (https://www.flyrnai.org/snapdragon, accessed on 10 November 2022) to amplify target fragments of *SmHsp70A1-1* (393 bp), *SmHsp70A1-2* (221 bp), and the green fluorescent protein gene *GFP* (315 bp, nonspecific negative control). The resulting PCR products were used as templates for the in vitro transcription of dsRNA using the T7 RiboMAX Express RNAi system (Promega, Madison, WI, USA), following the provided instructions. The synthesized dsRNA was checked by electrophoresis on 1% agarose gels and quantified using the BioSpec-nano spectrophotometer (Shimadzu, Kyoto, Japan). Before storage, the dsRNA was diluted to 10 μg/μL with RNase-free water and stored at −80 °C until further use.

For RNAi assays, cocooned larvae collected in January (post-diapause quiescent larvae) were selected due to their high levels of *SmHsp70A1-1* and *SmHsp70A1-2* expression at this particular stage (refer to Section 3). Initial in vitro assessments of gene knockdown efficiency and organismal viability following RNAi treatment led to the selection of the optimal delivery parameters as 30 nL of dsRNA solution at 10 μg/μL concentration. Accordingly, each test subject was microinjected with 30 nL dsRNA (300 ng) between the fifth and sixth abdominal segments using a Nanoject II microinjection device (Drummond Scientific Company, Broomall, PA, USA). The control groups received equivalent quantities of DEPC-water and ds*GFP* injections, respectively. Post-injection, the larvae were transferred to Petri dishes lined with moistened filter paper and were incubated under controlled conditions at 24 ± 1 °C and 70% ± 5% relative humidity (RH), with a 16:8 h light/dark photoperiod for 24 h. Twenty larvae per treatment group were sampled for qRT-PCR assessment of the gene knockdown efficiency, with three biological replicates per time point.

### 2.9. Effects of RNAI on Larval Cold Tolerance

To evaluate the effects of gene knockdown on cold tolerance in *S. mosellana* larvae, cocooned larvae (n = 3 replicates of 40 individuals each) were collected 24 h post-dsRNA injection and were exposed to −10 °C for 2 h in a controlled low-temperature incubator, as both target genes showed maximal expression at this temperature (refer to Section 3). After treatment, the larvae were returned to the controlled environmental conditions (24 ± 1 °C, 70 ± 5% RH, 16:8 light:dark cycle), and their status was monitored daily for six days to assess mortality rates. The larvae were classified as living only if they concurrently satisfied two conditions: (1) the ability to crawl after egress from the cocoon and (2) an observable response to gentle tactile stimulation when their motility was uncertain.

### 2.10. Data Analysis

All datasets were subjected to one-way ANOVA and Tukey’s post hoc analysis using SPSS 22.0 software (SPSS Inc., Chicago, IL, USA) to determine intergroup differences. Larval survival over time was assessed using the Kaplan–Meier estimator, with statistical comparisons conducted via the log-rank test in GraphPad Prism 9.5.0 (GraphPad Inc., San Diego, CA, USA). A significance threshold of *p* < 0.05 was applied for all statistical evaluations.

## 3. Results

### 3.1. Characterization of SmHsp70 Genes

**Figure 1:**
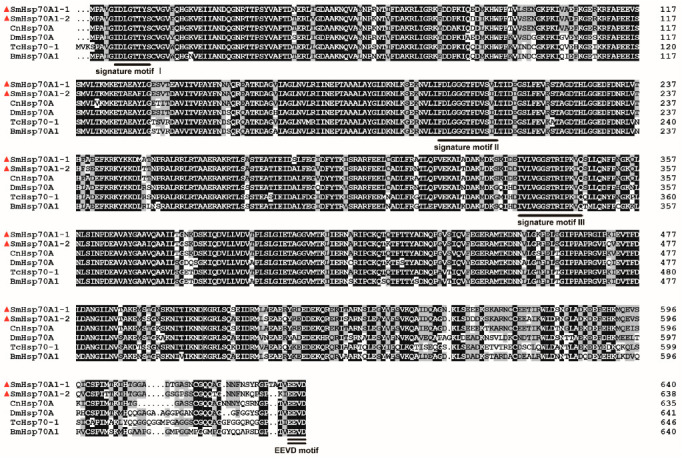
Sequence alignments of *Sitodiplosis mosellana* SmHsp70A1-1 (GenBank accession no. XXH62668.1) and SmHsp70A1-2 (XXH62669.1) with homologous proteins from other insect taxa, including *Contarinia nasturtii* (CnHsp70A, XP_031624417.1), *Drosophila melanogaster* (DmHsp70A, AAG26887.1), *Tribolium castaneum* (TcHsp70-1, QCI56580.1), and *Bombyx mori* (BmHsp70A, NP_001296526.1). SmHsp70s are marked by red triangles, with identical residues shown in black and similar ones in gray. Three signature motifs characteristic of the Hsp70 family are delineated with single underlining in black, and the cytosolic-specific consensus motif (EEVD) is emphasized with double underlining in black.

The ORFs of *SmHsp70A1-1* (GenBank accession no: PV849653) and *SmHsp70A1-2* (GenBank accession no: PV849654) obtained by PCR were found to be 1923 and 1917 bp in length (Figure S1), respectively, encoding polypeptides of 640 and 638 amino acids with predicted molecular masses of 70.45 and 70.02 kDa and theoretical isoelectric points of 5.45 and 5.49, respectively. The genomic DNA analysis showed that both genes lacked introns, while the encoded proteins shared three canonical Hsp70 signature motifs: IDLGTTYS (a.a. 7–13), IFDLGGGTFDVSIL (a.a. 194–206), and VVLVGGSTRIPKIQKS (a.a. 332–346) (Figure 1). In addition, the conserved cytosolic EEVD motif was identified at their C-termini (Figure 1). Subcellular localization predictions suggested cytoplasmic localization for both proteins.

Homology analysis indicated that SmHsp70A1-1 (XXH62669.1) and SmHsp70A1-2 (XXH62668.1) shared the highest degree of sequence identity with *Contarinia nasturtii* Hsp70A (XP_031624417.1), with 92% and 90% identity, respectively, and 78–82% identity with *Bombyx mori* Hsp70A1 (NP_001296526.1), *Tribolium castaneum* Hsp70-1 (QCI56580.1), and *Drosophila melanogaster* Hsp70A (AAG26887.1). The two SmHsp70s were 89% identical (Figure 1).

**Figure 2:**
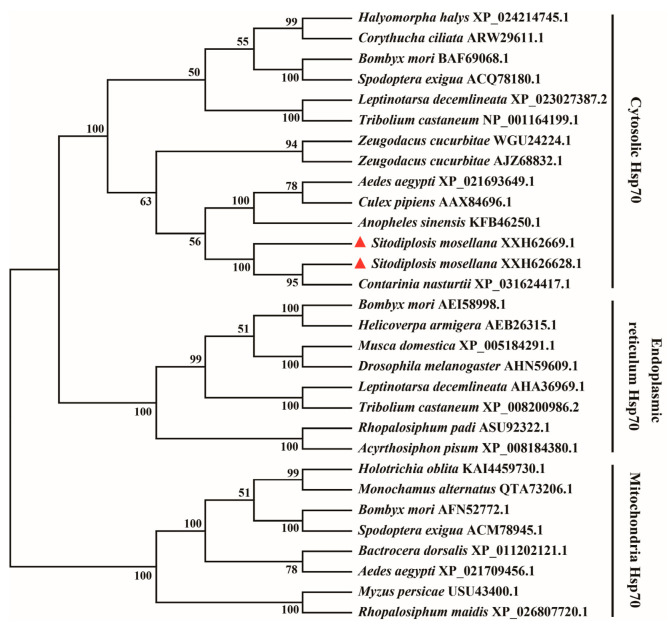
Phylogenetic tree of SmHsp70A1-1 and SmHsp70A1-2 (red triangles) with other known insect Hsp70s based on the neighbor-joining method.

The phylogenetic analysis revealed that insect Hsp70s were segregated into three primary clades corresponding to specific subcellular localizations, namely, cytosol, endoplasmic reticulum, and mitochondria (Figure 2). SmHSP70A1-1 and SmHsp70A1-2 were clustered into the cytosolic clade and were most closely related to Hsp70 from *C. nasturtii* (Figure 2).

### 3.2. Expression Patterns of SmHsp70 Genes at Different Developmental Stages

**Figure 3:**
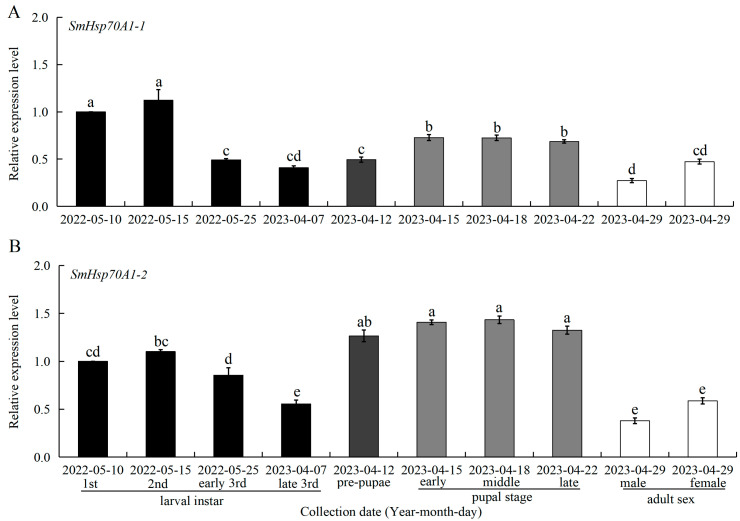
Expression patterns of *SmHsp70A1-1* (**A**) and *SmHsp70A1-2* (**B**) at different developmental stages. Relative mRNA levels (mean ± SE) at different developmental stages are quantified against the first-instar larvae (value = 1). Different letters denote statistically significant differences at each developmental stage (Tukey’s multiple range test, *p* < 0.05).

The transcription patterns of *SmHsp70A1-1* and *SmHsp70A1-2* were determined in *S. mosellana* at various developmental stages, including 1st- to 3rd-instar larvae, pre-pupae, early- to late-stage pupae, and adult males and females (Figure 3). The expression levels of *SmHsp70A1-1* peaked in 1st- and 2nd-instar larvae, followed by the pupal stage, with no significant difference observed between the 3rd-instar larvae and pre-pupae (Figure 3A). In contrast, *SmHsp70A1-2* expression in 1st- and 2nd-instars was lower than in pre-pupae and pupae but higher than that in adult males and females (Figure 3B).

### 3.3. Expression Patterns of SmHsp70 Genes During Diapause

**Figure 4:**
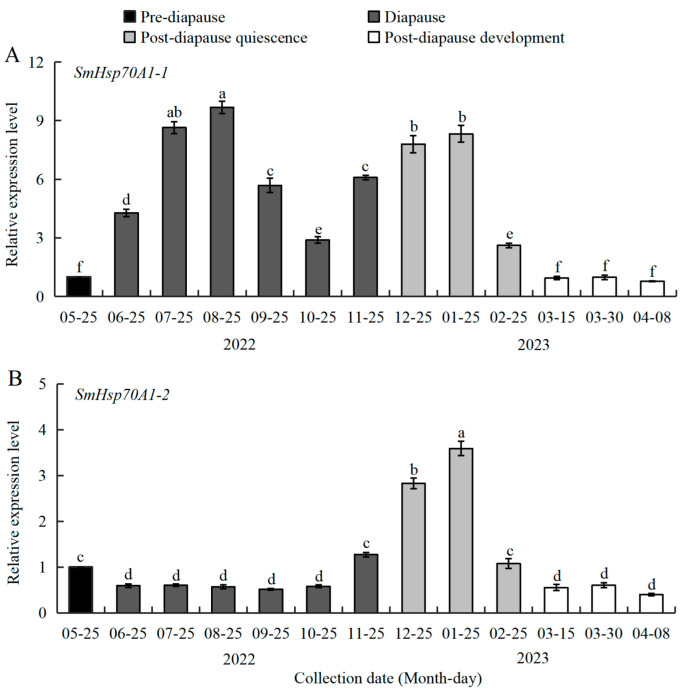
Expression patterns of *SmHsp70A1-1* (**A**) and *SmHsp70A1-2* (**B**) in pre-diapausal, diapausal, and post-diapausal larvae of *Sitodiplosis mosellana*. Relative mRNA levels (mean ± SE) at different diapause stages are quantified against pre-diapausing larvae (value = 1). Different letters denote statistically significant differences at different diapause stages (Tukey’s multiple range test, *p* < 0.05).

To clarify the association between diapause and *SmHsp70A1-1*/*SmHsp70A1-2* expression, the mRNA transcriptional abundance of both genes was quantified in 3rd-instar *S. mosellana* larvae across four diapause-associated physiological stages: pre-diapause, diapause, post-diapause quiescence, and post-diapause development (Figure 4). *SmHsp70A1-1* exhibited relatively low transcript abundance at the pre-diapause phase (May), with significant upregulation at diapause entry (June). High expression was maintained throughout diapause (June–November) and post-diapause quiescence (December to the following February), followed by a rapid decline to baseline pre-diapausal levels after the onset of post-diapausal development (March to early April in the following spring). The apex of expression was observed during July and August (summer), with a secondary peak in December and January (winter) (Figure 4A).

In contrast, the expression of *SmHsp70A1-2* exhibited significant downregulation at the start of diapause, with persistent suppression during diapause (June–October), followed by marked upregulation from late November as the populations entered post-diapause quiescence [33]. The expression levels reached their peak during mid-quiescence (January) before rapidly declining to diapause-equivalent levels during the post-diapausal developmental phases (Figure 4B).

### 3.4. 20E Regulation of SmHsp70 Genes During Diapause

**Figure 5:**
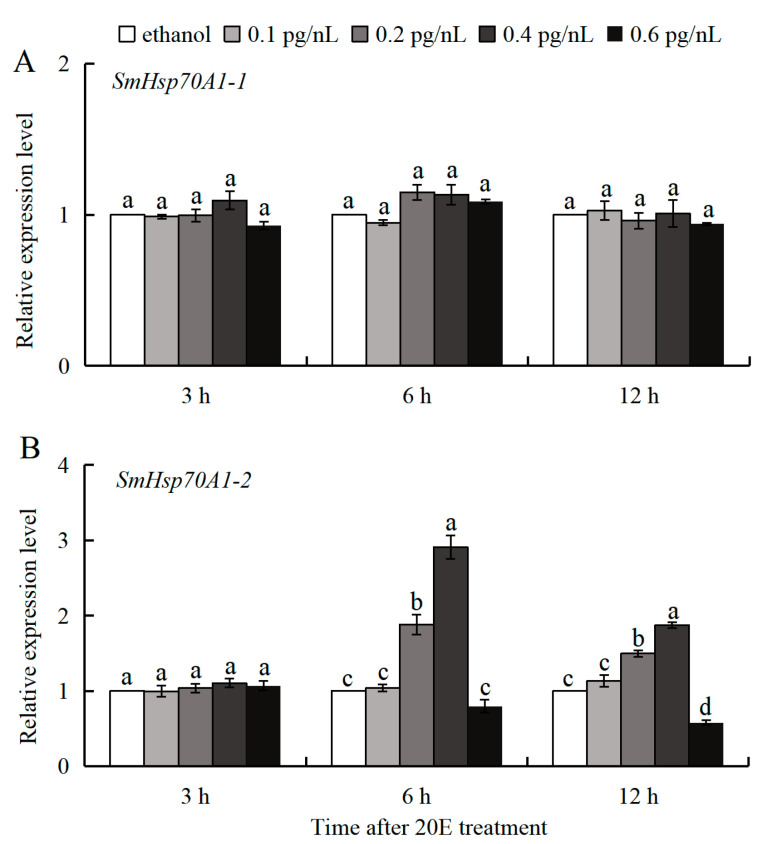
Expression patterns of *Hsp70A1-1* (**A**) and *Hsp70A1-2* (**B**) at 3, 6, and 12 h post-injection of 20E (0.1–0.6 pg/nL) into October-collected diapausing larvae of *Sitodiplosis mosellana*. Relative mRNA levels (mean ± SE) at each time point are quantified against the ethanol control (value = 1). Different letters denote statistically significant differences at each time point (Tukey’s multiple range test, *p* < 0.05).

The distinct expression patterns of the two *SmHsp70s* were observed in the diapausing larvae collected during October, which had been treated with 20E (0.1–0.6 pg/nL). The expression of *SmHsp70A1-1* remained unchanged, while that of *SmHsp70A1-2* exhibited significant dose-time-dependent induction at 6 and 12 h post-treatment (Figure 5). At 0.2 and 0.4 pg/nL of 20E, *SmHsp70A1-2* expression increased by 1.88- and 2.91-fold at 6 h and by 1.50- and 1.87-fold at 12 h, respectively, compared to the control (ethanol) group (Figure 5B). No significant changes in expression were detected for either gene at 3 h post-injection at any of the tested 20E concentrations (Figure 5A,B).

### 3.5. Patterns of SmHsp70 Gene Expression in Response to Heat and Cold Temperature Stress During Diapause

**Figure 6:**
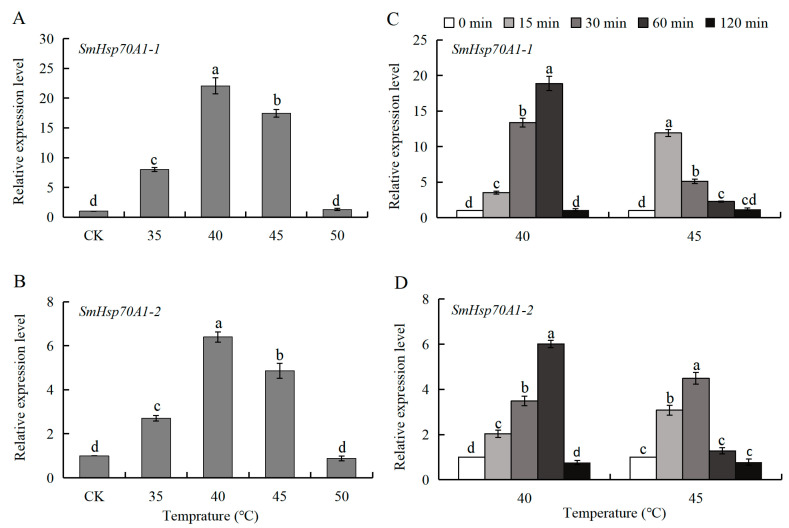
Heat-induced expression profiles of *Sitodiplosis mosellana Hsp70A1-1* and *Hsp70A1-2* in over-summering diapause larvae treated with various high temperatures (35–50 °C) for 1 h (**A**,**B**) or 40–45 °C for varying durations (0–120 min) (**C**,**D**). Relative mRNA levels (mean ± SE) at each treatment are quantified against the untreated control (CK, value = 1). Different letters denote statistically significant differences (Tukey’s multiple range test, *p* < 0.05).

The heat-responsive expression patterns of *SmHsp70A1-1* and *SmHsp70A1-2* were very similar in the over-summering diapause larvae. When compared to the untreated group (CK), both *SmHsp70A1-1* and *SmHsp70A1-2* exhibited pronounced upregulation at temperatures between 35 °C and 45 °C, reaching a maximum at 40 °C, with 22.07-fold and 6.39-fold increases, respectively. No notable changes in the expression of either gene were observed at 50 °C (Figure 6A,B).

The effects of varying exposure durations at 40 °C and 45 °C on the mRNA expression profiles of both genes were then investigated. Transcriptional upregulation of both genes was observed after exposure to heat stress for 15 to 60 min at 40 °C, with peak expression observed at 60 min. At 45 °C, the highest gene expression levels occurred between 15 and 30 min, with *SmHsp70A1-1* reaching peak transcription at 15 min and *SmHsp70A1-2* at 30 min (Figure 6C,D).

**Figure 7:**
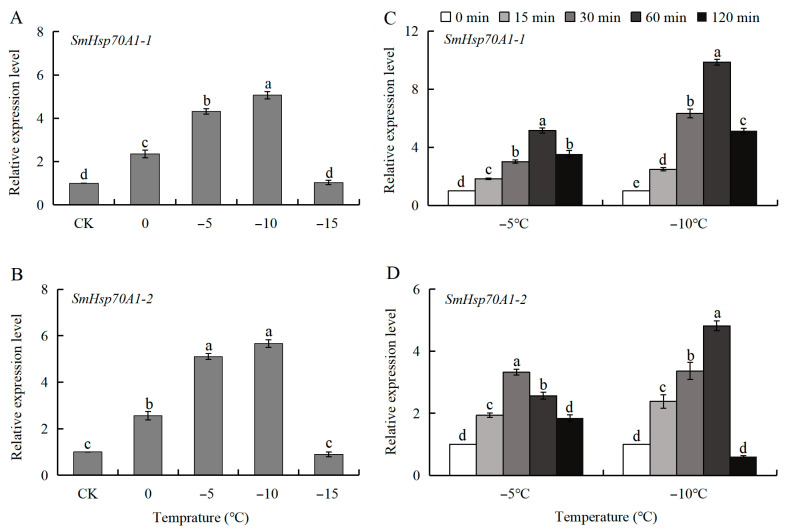
Cold-induced expression profiles of *Sitodiplosis mosellana Hsp70A1-1* and *Hsp70A1-2* in over-wintering diapause larvae treated with various low temperatures (0–−15 °C) for 1 h (**A**,**B**) or −5–−10 °C for varying durations (0–120 min) (**C**,**D**). Relative mRNA levels (mean ± SE) at each treatment are quantified against the untreated control (CK, value = 1). Different letters denote statistically significant differences (Tukey’s multiple range test, *p* < 0.05).

Similarly, the transcriptional expression levels of *SmHsp70A1-1* and *SmHsp70A1-2* were quantified in over-wintering larvae after an hour of exposure to different low temperatures. Both genes showed significant cold-induced expression above −15 °C, reaching maximum expression at −10 °C, with an 8.53-fold increase in *SmHsp70A1-1* levels and a 5.66-fold increase in *SmHsp70A1-2* levels relative to the control group (Figure 7A,B).

### 3.6. Silencing of SmHsp70 Genes by RNAI and Assessment of Susceptibility to Cold Stress

**Figure 8:**
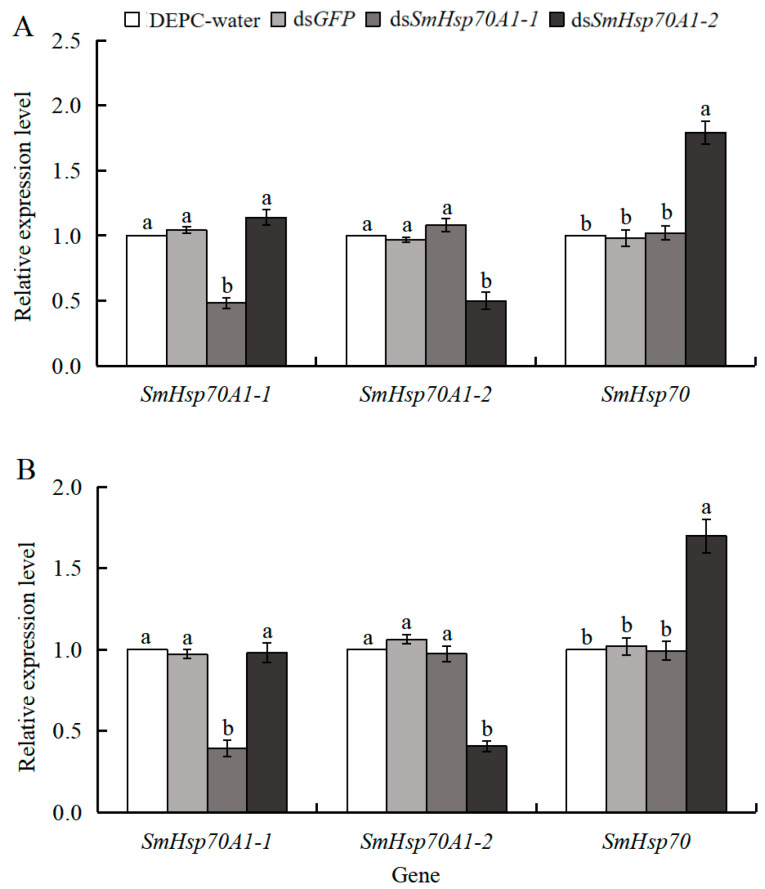
Expression patterns of three *SmHsp70s* at 24 h (**A**) and 48 h (**B**) post-dsRNA injection. Relative mRNA levels (mean ± SE) at each gene are quantified against the DEPC-water control (value = 1). Data are presented as means (n = 3) ± standard error (SE). Different letters indicate statistically significant differences (Tukey’s multiple range test, *p* < 0.05).

To assess the efficacy of RNAi-mediated gene silencing, the expression of *SmHsp70A1-1*, *SmHsp70A1-2*, and *SmHsp70* (GenBank: KJ813013) was determined after dsRNA injection. Significant knockdown of *SmHsp70A1-1* and *SmHsp70A1-2* was observed at 24 h and 48 h post-injection relative to the DEPC-treated water and ds*GFP* controls. Specifically, 24 h after injection with ds*SmHsp70A1-1*, the expression of *SmHsp70A1-1* had decreased by 52% and 56% compared to the DEPC-treated water and ds*GFP* control, respectively, whereas that of *SmHsp70A1-2* and *SmHsp70* remained unaffected. After injection with ds*SmHsp70A1-2*, the expression levels of *SmHsp70A1-2* were reduced by 50% and 52%, respectively, compared to the DEPC-treated water and ds*GFP* controls. Although no effect on *SmHsp70A1-1* was observed, *SmHsp70* expression was upregulated by 79% and 83% compared to DEPC-treated water and ds*GFP* control, respectively (Figure 8A).

Similarly, 48 h after ds*SmHsp70A1-2* injection, respective reductions of 59% and 61% were observed in *SmHsp70A1-2* expression compared to the DEPC-treated water and ds*GFP* controls, with no effects on *SmHsp70A1-1* and 70% and 73% increases in *SmHsp70* expression, respectively. After ds*SmHsp70A1-1* administration, the expression of *SmHsp70A1-1* decreased by 61% and 60%, respectively, relative to the DEPC-treated water and ds*GFP* controls, whereas that of *SmHsp70A1-2* and *SmHsp70* remained unchanged (Figure 8B).

**Figure 9:**
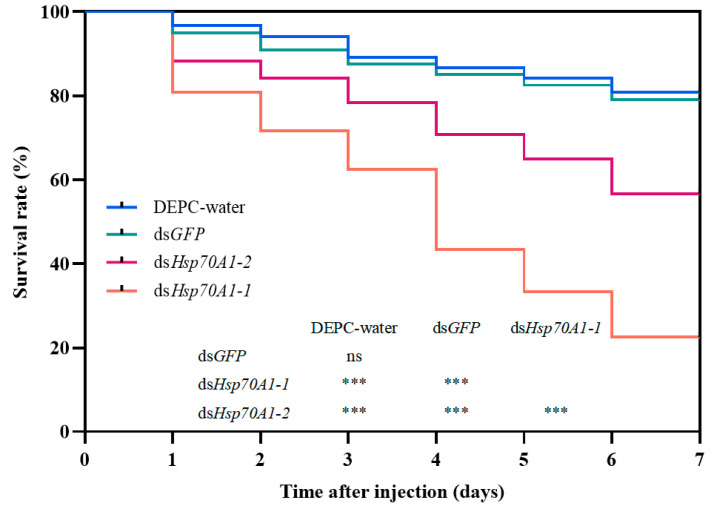
Kaplan–Meier survival curves of *Sitodiplosis mosellana* larvae after dsRNA injection. Different Kaplan–Meier survival curves correspond to different experimental groups (n = 120 per group), with DEPC-water as the blank control and ds*GFP* as the nonspecific dsRNA control. Statistical comparisons were performed using log-rank tests, with significance levels indicated as ***, *p* < 0.001; ns, *p* > 0.05.

Twenty-four hours after knockdown, the larvae were subjected to a −10 °C cold shock for 2 h in a controlled low-temperature incubator, followed by monitoring of subsequent mortality for six days. The mortality rates of the *SmHsp70A1-1* and *SmHsp70A1-2*-silenced larvae were found to increase significantly compared to the DEPC-water or ds*GFP*-treated groups. Each experimental cohort (DEPC-water, ds*GFP*, ds*Hsp70A1-1*, and ds*Hsp70A1-2*; n = 120 per group) showed cumulative six-day mortality rates of 19% (23/120), 22% (26/120), 78% (93/120), and 43% (52/120), respectively. Furthermore, the mortality rate in the *SmHsp70A1-1*-silenced group was significantly higher than that observed in the *SmHsp70A1-2*-silenced group. These findings indicate that the suppression of *SmHsp70A1-1* and *SmHsp70A1-2* significantly increased susceptibility to cold stress in *S. mosellana* larvae (Figure 9).

## 4. Discussion

Members of the Hsp70 family are pivotal components of the molecular chaperone network in cells [39]. The number of *Hsp70* genes varies according to the insect species [2,40,41]. In *S. mosellana* larvae, one *Hsp70* had been previously identified and was believed to play a crucial role in thermal stress responses during diapause [34]. Here, we isolated two additional *Hsp70* transcripts (*SmHsp70A1-1* and *SmHsp70A1-2*) from this species, which shared high sequence homology with orthologs in other taxonomic groups. The comparison of the cDNA and genomic DNA nucleotide sequences indicated that the two genes are intronless. The absence of introns is posited to enhance the expression of stress-responsive genes, as the lack of mRNA splicing enables rapid accumulation of the transcripts and subsequent responses to stress [19,42].

As anticipated, each deduced SmHsp70 protein sequence contained three Hsp70 family signature motifs, namely, IDLGTTYS, FDLGGGTFDVSV/IL, and IVLVGGSTRIPKV/IQ, which are distinctive hallmarks of this family (Figure 1) [43,44]. In addition, highly conserved EEVD motifs, shown to be essential for chaperone-binding activity [45,46], were identified in the C-terminal regions of both SmHsp70s (Figure 1), suggesting their localization within the cytoplasm. Predictions of subcellular localization and phylogenetic analyses demonstrated the classification of the two SmHsp70s within cytosolic subfamilies (Figure 2). While the SmHsp70s showed extensive regions of conservation, greater variability was observed in the C-terminal domains, suggesting that these differences may determine the functional specificities of individual Hsps [47,48].

Insect Hsps have been found to be associated with the regulation of insect growth, development, and metamorphosis [18,49]. Typically, the expression levels of most insect Hsps vary considerably across different developmental stages within a species. For instance, *TcHsp70III* in *Tribolium castaneum* is highly expressed in adults and young larvae but at lower levels in older larvae and pupae [50], while in *Galeruca daurica*, *GdHsp70* exhibits elevated expression in eggs and first-instar larvae [51]. In the present study, the highest levels of *SmHsp70A1-1* were observed in the first- and second-instar larvae, followed by the pupae, whereas *SmHsp70A1-2* exhibited its highest expression during the pre-pupal and pupal stages (Figure 3). These developmental stages correspond to periods of elevated basal metabolic activity, as feeding occurs during the early larval stage, while metamorphosis is associated with the pupal stage. Similar developmental stage-preferred expression patterns have been documented in *Cydia pomonella* [1], *Nilaparvata lugens* [52], and *Myzus persicae* [53]. Moreover, the pupal stage involves the degradation and reconstruction of multiple organs and tissues, which may contribute to the increased expression of *Hsps*, as observed with *Hsp70* in *Grapholitha molesta* [54] and *Hsp71.3* in *Glyphodes pyloalis* [55].

As described in the Introduction section, many studies have shown that the roles of *Hsp70s* in mediating diapause vary considerably across different insect taxonomic groups. The present research on *S. mosellana* revealed pronounced upregulation of *SmHsp70A1-1* during diapause, especially in summer and winter (Figure 4A), consistent with the expression patterns of *SmHsp70/90* [34]. The current evidence suggests that diapause-induced Hsps may directly induce cell cycle arrest [56], as exemplified in *Drosophila melanogaster*, where elevated levels of small *Hsps* and *Hsp70* are correlated directly with cell cycle arrest or retardation [57,58]. It is possible that *Hsp70A1-1* in *S. mosellana* may also have similar regulatory roles during diapause, although this specific role warrants further molecular investigation.

In contrast to *SmHsp70A1-1*, *SmHsp70A1-2* expression was found to be downregulated upon entry into diapause but increased markedly during the shift to post-diapause quiescence (Figure 4B), correlating with fluctuations in 20E titers documented in this species [36]. Functional crosstalk has been reported between 20E signaling pathways and heat shock protein regulatory mechanisms [59,60]. In *Bombyx mori Bm12* cell lines, 20E induces the expression of *Hsp70s*, which enhances 20E signal transduction by promoting EcR nuclear localization [18]. The observed 20E-mediated transcriptional induction of *S. mosellana Hsp70A1-2* in diapausing larvae provides additional evidence of the linkage between 20E signaling and *Hsp70A1-2* expression (Figure 5B). This pronounced upregulation at the transition of diapause to post-diapause quiescence suggests that *SmHsp70A1-2* may represent a dependable molecular indicator for predicting this developmental shift.

Temperature has a profound influence on insect dispersal and population dynamics. Extreme temperatures lead to increased synthesis of Hsps in insects as an adaptive mechanism [19,61]. Similar to patterns observed in *Bemisia tabaci* for *Hsp70* [43], *Bombyx mori* for *Hsp70-1*, *Hsp70-2*, and *Hsp70-3* [62], and *Pieris melete* for *Hsp70a* and *Hsp70b* [21], the two *S. mosellana Hsp70* transcripts were significantly induced by heat and cold stress within a brief timeframe during larval diapause, although this induction became less pronounced with prolonged exposure and further increases or decreases in temperature (Figure 6 and Figure 7). Notably, exposure to 47.5 °C for 1 h was found to be lethal to cocooned *S. mosellana* larvae [63], indicating the limitations of Hsp-mediated thermoprotection under extreme heat stress. This may explain why agronomic practices that elevate soil temperature are effective in pest management; during the fallow summer period, increased solar radiation is absorbed by the surface soils, while tillage redistributes the larvae into the warmer upper-soil strata, with both factors contributing to pest mortality [64,65].

In general, the upregulation of *Hsp* gene transcription in insects is closely associated with enhanced tolerance to low-temperature stress [19,66]. For instance, in the flesh fly *Sarcophaga crassipalpis*, *Hsp70* exhibits a developmentally upregulated response during the over-wintering pupal diapause [67]. While RNAi inhibition of *Hsp70* upregulation in response to cold in *S. crassipalpis* did not affect diapause initiation or duration, it significantly reduced low-temperature tolerance in the pupae [22]. Similarly, *Spodoptera exigua* larvae exhibited increased sensitivity to temperature around 0 °C and higher mortality after Hsp70-specific dsRNA injection [17]. In this study, the knockdown of *SmHsp70A1-1* and *SmHsp70A1-2* significantly reduced the survival rate of *S. mosellana* larvae at −10 °C, indicating that both genes confer protective effects that enhance cold tolerance in this pest. Notably, larval mortality was significantly higher in the *SmHsp70A1-1*-knockdown group compared to the *SmHsp70A1-2* group at −10 °C (Figure 9), suggesting compensatory effects, as the suppression of *SmHsp70A1-2* resulted in a significant upregulation of *SmHsp70* expression (Figure 8), and vice versa (Figure S2). However, due to the lack of specific monoclonal antibodies against SmHsp70A1-1 and SmHsp70A1-2, this study was limited to mRNA-level analysis after gene knockout; thus, protein-level verification post-knockout requires further investigation.

## 5. Conclusions

The study findings demonstrate that both *SmHsp70A1-1* and *SmHsp70A1-2* are developmentally and environmentally regulated. The expression of *SmHsp70A1-1* was upregulated during diapause, whereas that of *SmHsp70A1-2* showed downregulation and dose-dependent induction by 20E. Their induction in response to heat (35–45 °C) and cold (0–−10 °C) stress, as well as the cold sensitivity induced by gene-specific RNA interference, indicated that both genes play crucial roles in conferring resistance or tolerance to environmental stresses in *S. mosellana*. Further investigation into more *SmHsp70s* and their interactions is essential for elucidating the adaptive mechanisms of *S. mosellana* and promoting efficient integrated pest management under environmental selection pressures.

## Figures and Tables

**Table 1:** Primer sequences used in this study.

Primer Name	Sequence (5′ to 3′)	Purpose
Hsp70A1-1 sense	GCGAAAGAAAGGAGGAGAAG	ORF and gDNA cloning
Hsp70A1-1 antisense	CAGGCTCTCTTATTGTACGAG	
Hsp70A1-2 sense	ATGCCTGCAGTTGGAATTG	
Hsp70A1-2 antisense	GCAAAGAGTGATTTCTTCCTCG	
dsHsp70A1-1 sense	taatacgactcactatagggAAAACCAAGTCGCCATGAAC	dsRNA synthesis
dsHsp70A1-1 antisense	taatacgactcactatagggTATCCAATCCGTAGGCCAAG	
dsHsp70A1-2 sense	taatacgactcactatagggGAAGGCGAACGAGCTATGAC	
dsHsp70A1-2 antisense	taatacgactcactatagggCGATCGATTTCTGCTTGTGA	
dsGFP sense	taatacgactcactatagggGTGTTCAATGCTTTTCCCGT	
dsGFP antisense	taatacgactcactatagggCAATGTTGTGGCGAATTTTG	
Hsp70A1-1 sense	AATCAACCCAGACGAGGCAG	qPCR
Hsp70A1-1 antisense	GCCCAATGAGAGTGGTGTCA	
Hsp70A1-2 sense	CGTAATTGCTGCGAGGAAGC	
Hsp70A1-2 antisense	GTGATTGGCGAGCAAACCTG	
GAPDH sense	CCATCAAAGCAAGCAAGA	
GAPDH antisense	CAGCACGGAGCACAAGAC	

## Data Availability

The original contributions presented in this study are included in the article/Supplementary Material. Further inquiries can be directed to the corresponding author(s).

## Supplementary Materials

The following supporting information can be downloaded at: https://www.mdpi.com/article/10.3390/biology14091147/s1, Figure S1. Nucleotide and deduced amino acid sequences of *SmHsp70A1-1* and *SmHsp70A1-2* in *Sitodiplosis mosellana*. Initiation codons (ATG) and termination codons (TGA/TAA) were marked with ellipses. Three signature motifs characteristic of the Hsp70 family were highlighted by shading, and the cytosolic-specific consensus motif (EEVD) was boxed, Figure S2. Expression patterns of three *SmHsp70s* at 24 h (A) and 48h (B) after ds*SmHsp70* injection. Relative mRNA levels (mean ± SE) at each gene are quantified against the DEPC-water control (value = 1). Different letters indicate statistically significant differences (Tukey’s multiple range test, *p* < 0.05).
