# Supplementary material for: Characterization of Two Novel Heat Shock Protein 70 Transcripts from Sitodiplosis mosellana and Their Response to Larval Diapause and Thermal Stress
Source: Biology (Basel). 2025 Aug 30;14(9):1147. doi: 10.3390/biology14091147 (PMC12467714; doi:10.3390/biology14091147)
Supplement: Supplementary file 1 [file biology-14-01147-s001.zip › biology-3834453-supplementary.pdf]

Supplementary Materials: The following are available online. Figures S1 and S2.

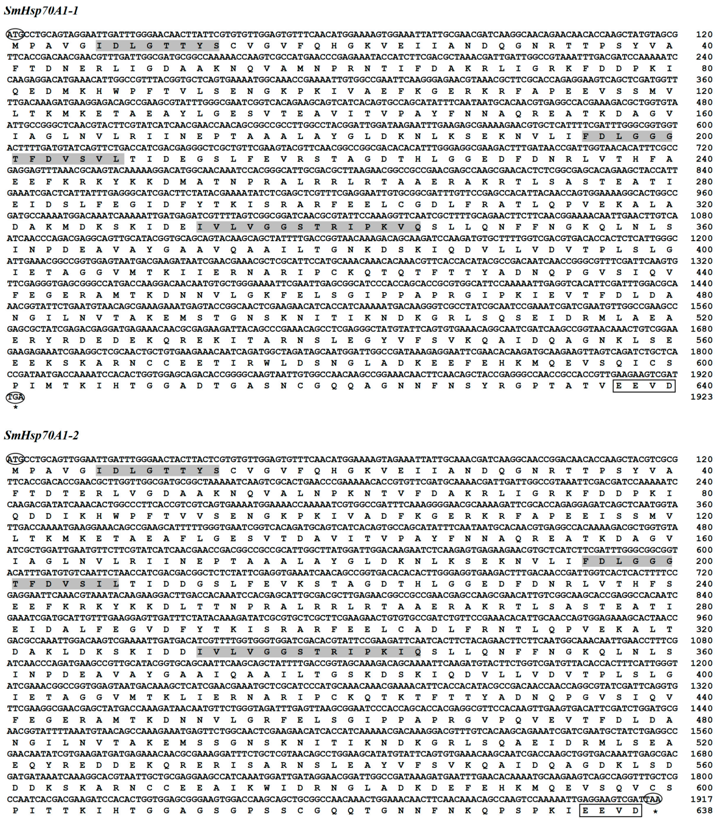

Figure S1. Nucleotide and deduced amino acid sequences of *SmHsp70A1-1* and *SmHsp70A1-2* in *Sitodiplosis mosellana*. Initiation codons (ATG) and termination codons (TGA/TAA) were marked with ellipses. Three signature motifs characteristic of the Hsp70 family were highlighted by shading, and the cytosolic-specific consensus motif (EEVD) was boxed.

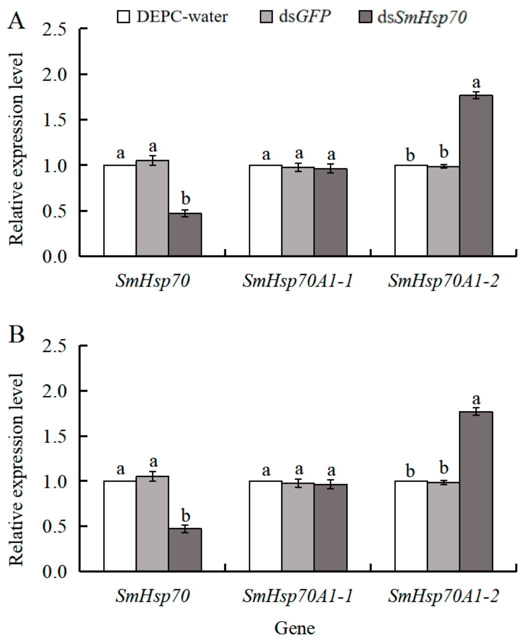

Figure S2. Expression patterns of three *SmHsp70s* at 24 h (A) and 48h (B) after ds*SmHsp70* injection. Relative mRNA levels (mean ± SE) at each gene are quantified against the DEPC-water control (value = 1). Different letters indicate statistically significant differences (Tukey's multiple range test, $P < 0.05$).
